# Correction of scientific literature: Too little, too late!

**DOI:** 10.1371/journal.pbio.3001572

**Published:** 2022-03-03

**Authors:** Lonni Besançon, Elisabeth Bik, James Heathers, Gideon Meyerowitz-Katz

**Affiliations:** 1 Faculty of Information and Technology, Monash University, Clayton, Victoria, Australia; 2 Harbers Bik LLC, San Francisco, California, United State of America; 3 Cipher Skin, Denver, Colorado, United State of America; 4 School of Health and Society, University of Wollongong, Wollongong, New South Wales, Australia

## Abstract

Four scientists who have identified issues in published papers struggled to get them amended or retracted; here they present evidence that these struggles are widely shared and that scientific advances are presently critically hindered. They make suggestions for academic actors that would help scientists raise concerns about submissions and improve academia’s correction rate and speed.

## Teaser

The Coronavirus Disease 2019 (COVID-19) pandemic has highlighted the limitations of the current scientific publication system, in which serious post-publication concerns are often addressed too slowly to be effective. In this Perspective, we offer suggestions to improve academia’s willingness and ability to correct errors in an appropriate time frame.

Traditionally, scientific progress has relied on trust and the relatively slow cycle of peer review, publication, and citation of research data. The current Coronavirus Disease 2019 (COVID-19) pandemic not only accelerated the speed of research but also brought to light some severe shortcomings of the scientific publication process, such as failures to quickly address errors or to catch and prevent scientific misconduct.

Within months of the Severe Acute Respiratory Syndrome Coronavirus 2 (SARS-CoV-2) virus being identified, disease progression to COVID-19, viral transmission routes, and treatment options were being carefully studied, and effective vaccines had begun development. This was one of the most impressive scientific achievements of the modern era [1]. While the goal of disease mitigation has been rightfully praised for its comparative speed, organization, and safety, the mechanics of the publication system that sustained it have been far from ideal. We believe it is true to say the response to COVID-19 has succeeded in spite of, rather than because of, the present publication system.

During the COVID-19 pandemic, many basic quality control and transparency principles have been violated on a regular basis [2]. This is perhaps most apparent in the Surgisphere debacle [3], in which global policy on COVID-19 treatment was changed overnight on the basis of a database that later turned out not to exist. Although the Surgisphere retraction happened quickly, it was far slower than the change in medical practice, which was immediate, and represents a best-case scenario in which a high-profile paper was immediately interrogated and investigated. The stories of hydroxychloroquine and ivermectin, both widely promoted based on poor quality or even fraudulent studies [4], are further concerning accounts of how the scientific publishing process has failed to exercise basic quality control.

In the usual course of scientific investigation, these stories would be something of a footnote—fraud, malfeasance, and mendacity within median research studies are interesting to meta-scientists, methodologists, and theoreticians, but rarely have an impact outside of the scientific community. By contrast, high-profile studies with global implications receive a great deal of collective attention and scrutiny if they are untrustworthy, which normally limits their influence. However, in the setting of the present pandemic, poor research has sometimes been instantly applied to health policy after being published or simply publicized, and used in treatment regimens shortly after. There is an immediacy to the impact of scientific papers that was rarely present prior to the pandemic.

Traditional responses to such research issues (such as post-review letters, notes of concern, and even retractions) are woefully inadequate to address these problems. Nowadays, preprints and peer-reviewed research papers are rapidly shared on online platforms among millions of readers within days of being published. A paper can impact worldwide health and well-being in a few weeks online; that it may be retracted at some point months in the future does not undo any harm caused in the meantime. Even if a paper is removed entirely from the publication record, it will never be removed from the digital space and is still likely to be cited by researchers and laypeople alike as evidence. Often, its removal contributes to its mystique. For example, a retracted and proven false study on vitamin D from 2020 [5] that was pulled from the preprint server it was hosted on was still being cited uncritically as recently as November 2021.

All of these issues are compounded by the glacial pace at which scientific correction is likely to occur [6]. Identifying flaws in a paper may only take hours, but even the most basic formal correction can take months of mutual correspondence between scientific journal editors, authors, and critics. Even when authors try to correct their own published manuscripts, they can face strenuous challenges that prompt many to give up. Worse still, while editors and authors might gain financial and career benefits from ignoring errors, scientific critics are explicitly discouraged by the academic community from performing this work.

The authors of this Perspective have all been involved in error detection in this manner. For our voluntary work, we have received both legal and physical threats and been defamed by senior academics and internet trolls. While many scientists personally support error-checking work [7], academia as a whole seems to view error-checking as a dirty footnote to the achievement of publication. Even when papers are retracted, individuals who have spent endless hours explicating the problems therein are left with no formal career benefits whatsoever—if anything, they face substantial retaliation for correcting errors.

This system is unsustainable, unfair, and dangerous, and the pandemic has acted to magnify its unsuitability and numerous limitations. Rather than being a disappointing footnote, error-checking should be supported and funded by government agencies and research institutions. Public, open, and moderated review on PubPeer [8] and similar websites that expose serious concerns should be rewarded with praise rather than scorn, personal attacks, or threats (either legal or on the reviewers’ lives). Importantly, retraction should not always be seen as a failure. While some papers are retracted for reasons of serious research misconduct, other papers are retracted because of unintentional errors [9]. Scientists must acknowledge that any process comes with an error rate, and correcting mistakes should not limit careers, but instead enhance them [10]. Consequently, we propose some solutions that could improve the current error-checking and correction system in scientific publishing (Box 1).

**Box 1. Approaches to destigmatize and speed up the scientific correction process**

- Editors should issue an Expression of Concern within days after serious and verifiable concerns have been raised either privately or on a public forum. If the concerns are publicly raised, the Expression of Concern should link to them, otherwise, it should summarize the key points raised privately.
- Committee on Publication Ethics (COPE) guidelines for editors and journals should provide a timeline for responding to concerns about published papers, with, for instance, a maximum of 90 days to publicly highlight concerns, contact the authors, get a response from the authors, and publish it.
- Public, open, and post-publication peer review should be considered and rewarded by hiring and promotion committees as well as by funding bodies. Applications for funding or positions should consider such correction efforts made by scientists just as much as they consider the publication of new research results.
- To further establish an error-checking culture, scientists should be trained to recognize mistakes (including their own). Institutes and funding agencies should allocate time for error-checking, and institutions and journals should promote corrections and retractions as much as they promote new research findings.
- Notices of retractions or corrections could be linked to the researchers that initially raised concerns and attributed a DOI that links to their careful rebuttal of the original paper (even if it’s their own).
- Journal webpages should directly link to discussions on PubPeer rather than rely on the use of an external plug-in.
- DOI versioning already allows for concurrent versions of documents to coexist and should be adopted by publishing venues to allow for easy and fast correction of papers when needed, along with meta-data on the changes between versions.
- Critics who raise professional, nondefamatory concerns about a preprint or published paper should have explicit, paid legal protection (e.g., provided by their institutions or professional societies) against threats issued by the critiqued authors or their institutions.

Understandably, there are legal concerns when discussing the retraction and/or correction of scientific papers. We do not wish to minimize these issues, as they may be serious for both publishers and academics alike. However, many such problems stem from an environment in which retraction is seen as a career-ending calamity, and, thus, our proposed improvements (Box 1) may alleviate many of the legal concerns faced by the academic community.

Viewing the retraction and correction of scientific papers as a failure is a self-fulfilling prophecy: As the status of a paper rarely changes post-publication, it is seen as something exceptional or immoderate instead of a normal part of the scientific process. The alternative to addressing this problem is to continue to maintain a scientific commons that is unable to deal with the rapid dissemination and correction of research that is needed in the digital age.
